# Supplementary material for: DMRforPairs: identifying Differentially Methylated Regions between unique samples using array based methylation profiles
Source: BMC Bioinformatics. 2014 May 15;15:141. doi: 10.1186/1471-2105-15-141 (PMC4046028; doi:10.1186/1471-2105-15-141)
Supplement: Additional file 2 — DMRforPairs output for the comparison of A431-MCF7 and NA17018-NA17105. Please start from the HTML files in each folder. Available via the BMC Bioinformatics website. [file 1471-2105-15-141-S2.zip › 1394847754114233_MOESM2_ESM/A431_MCF7/figures/35279.pdf]

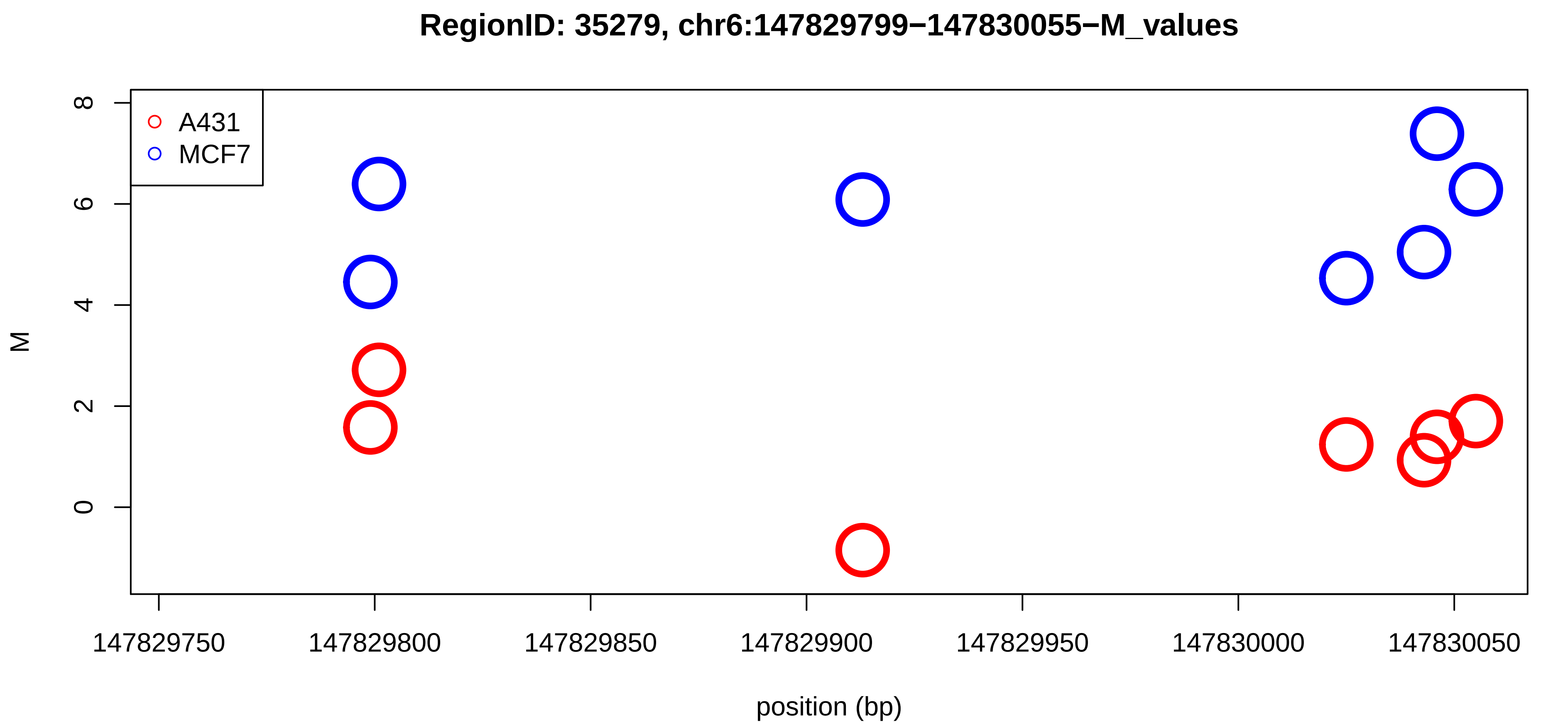
RegionID: 35279, chr6:147829799−147830055−M_values
A431
MCF7
M
0
2
4
6
8
147829750
147829800
147829850
147829900
147829950
147830000
147830050
position (bp)

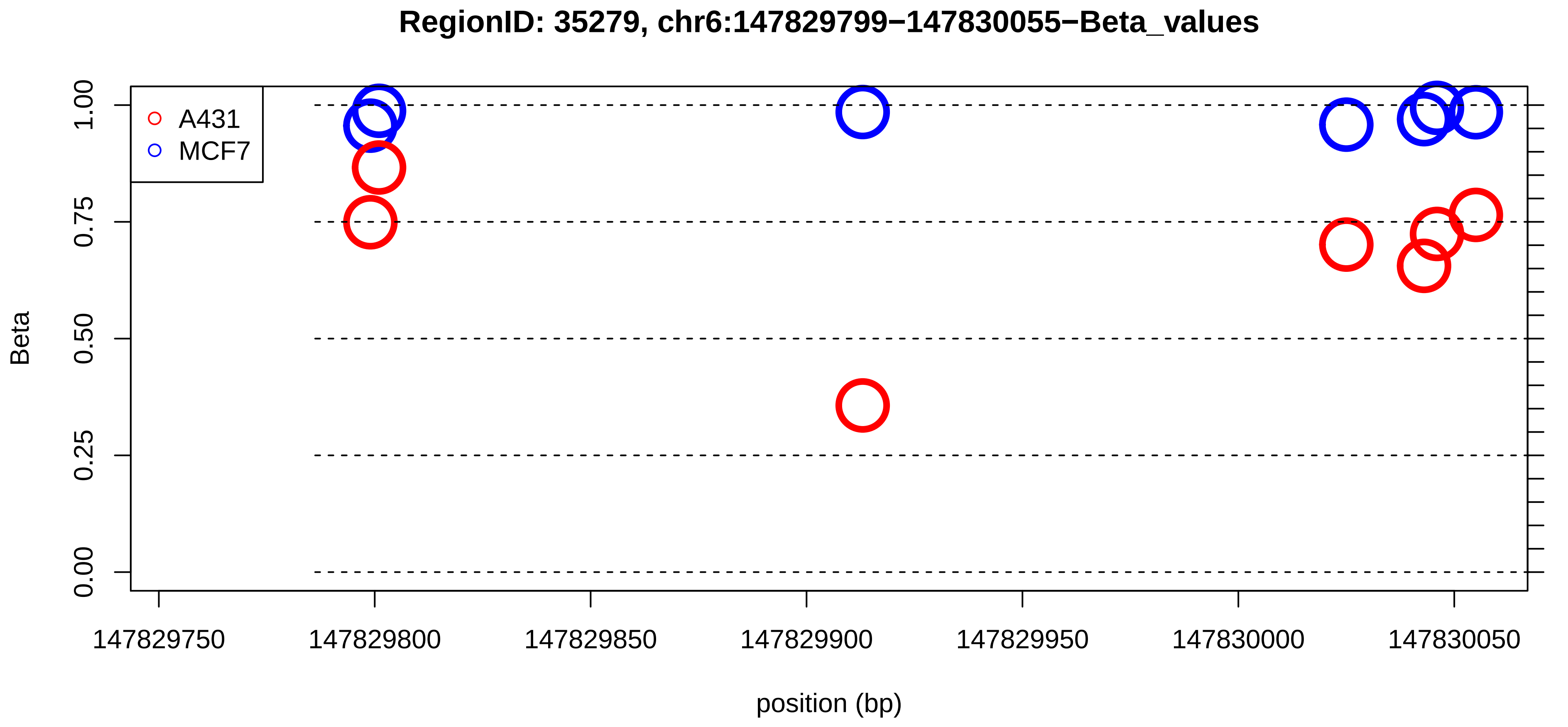
RegionID: 35279, chr6:147829799−147830055−Beta_values
A431
MCF7
Beta
0.00
0.25
0.50
0.75
1.00
147829750
147829800
147829850
147829900
147829950
147830000
147830050
position (bp)

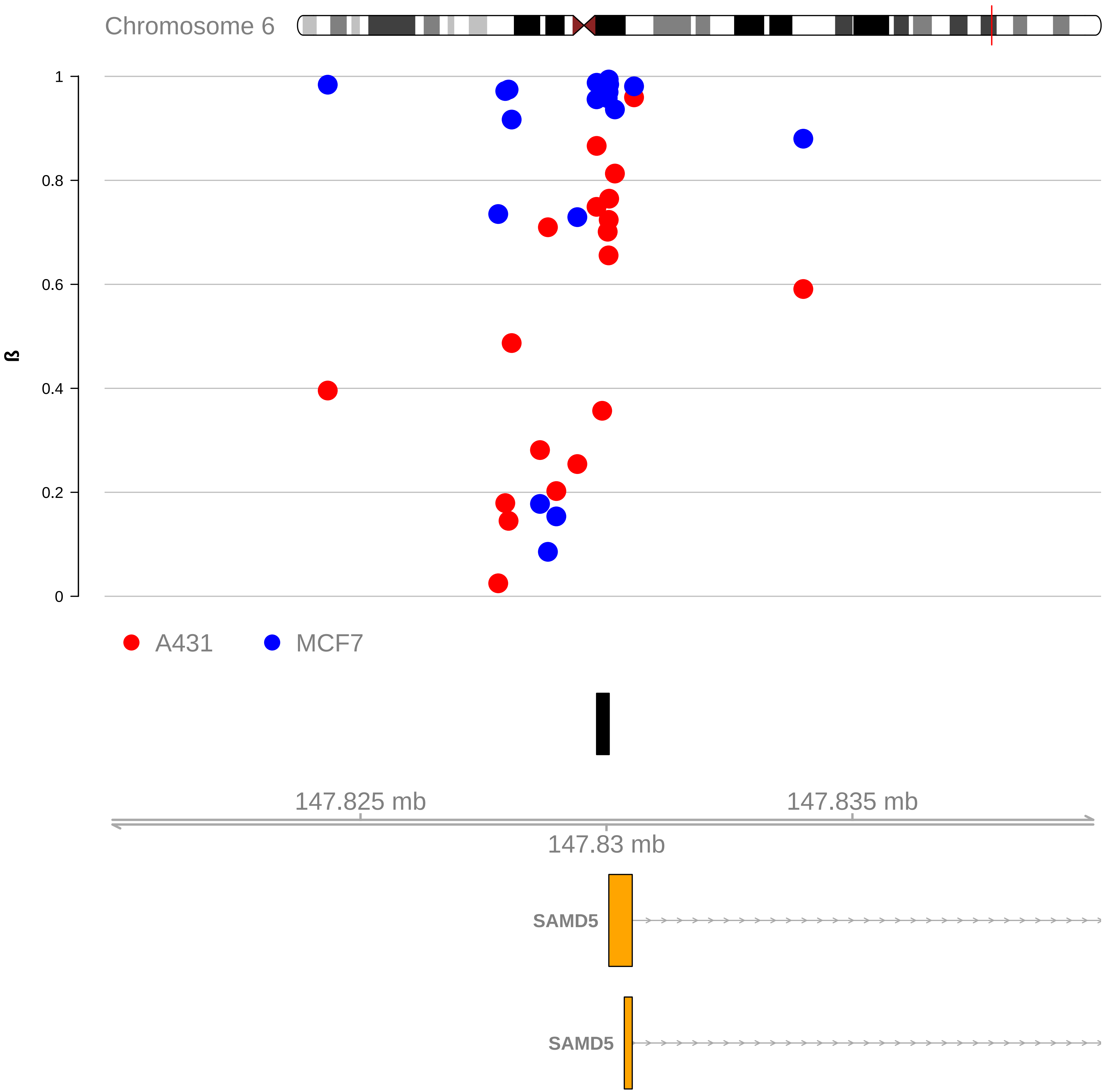
Chromosome 6
1
0.8
0.6
0.4
0.2
0
ß
A431
MCF7
147.825 mb
147.835 mb
147.83 mb
SAMD5
SAMD5
